# Successful Green Synthesis of Gold Nanoparticles using a *Corchorus olitorius* Extract and Their Antiproliferative Effect in Cancer Cells

**DOI:** 10.3390/ijms19092612

**Published:** 2018-09-03

**Authors:** Eman H. Ismail, Aliyah M. A. Saqer, Eman Assirey, Arshi Naqvi, Rawda M. Okasha

**Affiliations:** 1 Department of Chemistry, Taibah University, 30002 Al-Madinah Al-Munawarah, Saudi Arabia; dr_science_7@hotmail.com or eman_hamed@sci.asu.edu.eg (E.H.I.); MS-09life@hotmail.com (A.M.A.S.); eman_assirey@hotmail.com (E.A.); arshi_84@yahoo.com (A.N.); 2 Chemistry Department, Faculty of Science, Ain Shams University, 115566 Cairo, Egypt

**Keywords:** gold NPs, *Corchorus olitorius*, cytotoxicity, in-silico screenings

## Abstract

A facile bottom-up “green” synthetic route of gold nanoparticles (Au NPs) is described, using a leaf extract of the *Malvaceae* plant *Corchorus olitorius* as a reducing and stabilizing agent. The size and shape of the obtained nanoparticles were modulated by varying the amounts of the metal salt and the broth extract in the reaction medium. Only one hour was required for the complete conversion to Au NPs, suggesting that the reaction rate was higher or comparable to those of nanoparticles synthesized by chemical methods. The obtained nanoparticles were characterized by UV–visible spectroscopy, transmission electron microscopy (TEM), X-ray diffraction (XRD), Fourier-transform infrared (FTIR) spectroscopy, and thermal gravimetric analysis (TGA). While infrared spectroscopy was employed to characterize the various functional groups in the organic layer that stabilized the particles, TEM images were used to optimize the conditions for NPs growth. A low concentration of the *C. olitorius* extract yielded mixed triangular and hexagonal shapes; in contrast, quasi-spherical shapes of Au NPs with an average size of 37–50 nm were obtained at a higher extract broth concentration. The Au NPs displayed Surface Plasmon Resonance (SPR) bands at 535 nm. An in vitro cytotoxic assay of the biocompatible Au NPs revealed a strong cytotoxic activity in three human cancer cell lines, namely, colon carcinoma HCT-116, hepatocellular carcinoma HepG-2, and breast adenocarcinoma MCF-7. In-silico bioactivity, drug-likeness, and ADMET (Absorption, Distribution, Metabolism, Excretion, and Toxicity) predictions were conducted in order to examine the pharmacokinetic behavior of the compounds present in the *C. olitorius* extract.

## 1. Introduction

Over the past few decades, the possibility of developing new materials for biomedical applications has gradually increased because of the abundant advances made in the field of cancer diagnosis and therapy using nanoparticles (NPs). Nobel metal nanoparticles, such as gold (Au), platinum (Pt), and silver (Ag), are deemed as valuable precursors for the creation of innovative nanodevices and nanosystems that are constructed on the basis of a rational design with the precise integration of the tailored functional properties of NPs.

The unique Surface Plasmon Resonance (SPR) in gold nanoparticles triggers their photostability and extremely efficient absorptivity of light [1,2,3,4], thus offering multiple modalities for biological and medical applications, like X-ray computed tomography and magnetic resonance imaging [5], cancer nanotechnology [6], tissue engineering, drug and gene delivery [7], and fluorescent labeling [8].

The green synthesis of nanoparticles has attracted many researchers because of the high demand to produce clean, nontoxic chemicals, environmentally benign solvents, and renewable materials. Moreover, this methodology improves the biocompatibility of the obtained NPs [9,10,11]. For instance, many successful attempts have been reported in the literature, which achieve the synthesis of nanoparticles using biological systems, such as yeast, fungi, and bacteria [9,10,11,12].

Recently, the use of plant extracts has proven to be one of the most successful techniques for growing nanoparticles. Most of these studies employed broths, obtained from boiling fresh plant leaves [13,14,15,16,17,18,19]. For example, Au and Ag NPs have been prepared using several plant extracts as those of *Neem* leaves [13], *Hibiscus rosa sinensis* [14], *Ocimum tenuiflorum, Azadirachta indica* and *Mentha spicata* leaves, and *Citrus sinensis* [15], *Maduca longifolia* [16], and *Abelmoschus esculentus* peel [17]. So far, it has been established that the antioxidant components of the studied plant extracts are responsible for the reduction of metal salts, leading to the growth and stabilization of the NPs [13,14,15,16,17,18,19].

The plant extracts are generally mixtures containing a variety of compounds, including active component(s) [20,21]. In spite of the fact that each active constituent in herbal extracts claims efficacy, the combined components may reciprocally affect their respective pharmacokinetic behavior, thus hampering the extract’s safety and efficiency [21,22,23,24]. In-silico bioactivity, drug-likeness, and ADMET predictions [25,26] can help in prioritizing and assessing the pharmacokinetic properties of the predicted active constituents of plant extracts.

In this work, we are introducing a novel approach, using a *Corchorus olitorius* plant extract to produce Au NPs. *C. olitorius* is a culinary and medicinal herb from the *Tiliaceae* family which is widely used as a vegetable in several countries in Asia [27,28]. It is a popular Egyptian dish, which is also diffused in other Middle Eastern and North African countries. The leaves of this plant are rich in antioxidants, such as vitamin C, vitamin E, β-carotene, α-tocopherol, glutathione, and phenols [27,28]. The leaves also contain fatty acids, minerals, other vitamins, and mucilaginous polysaccharides [27,28]. Other studies have also demonstrated that *C. olitorius* exhibits anti-inflammatory and anti-proliferative activities in various in vitro and in vivo settings [29,30]. A recent report examined the use of a *C. olitorius* extract for the production of gold and iron oxide nanoparticles; however, the described procedure employed a high temperature to obtain the desired nanoparticles [31]. Herein, we succeeded in synthesizing Au nanoparticles at ambient temperature using the reduction power of a *C. olitorius* extract. The obtained nanoparticles were then examined for their therapeutic activity against three cancer cell lines. Moreover, an in-silico approach was undertaken to study the pharmacokinetic, toxicity, bioactivity, and drug-likeness profiles of the main constituents of the *C. olitorius* extract.

## 2. Results

A *C. olitorius* extract was prepared via boiling 8 g of dried mallow leaves in deionized water for 15 min. The obtained solution was filtered, and its volume was brought to 100 mL. For better results, the filtrate was kept in the dark at 10 °C and used within one week.

**Figure 1:**
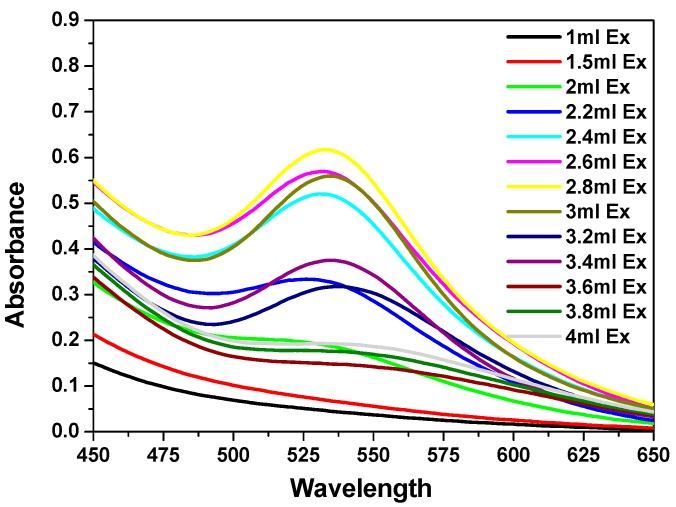
UV–visible spectra of Au nanoparticles (NPs) formed after 1 h in the presence of different volumes of *Corchorus olitorius* (mallow) leaf extract (1–3.4 mL) and 0.5 mL of (0.01 M) HAuCl_4_·3H_2_O.

The formation and stability of gold nanoparticles were followed by UV–visible spectrophotometry. Figure 1 shows the UV–vis spectra of gold nanoparticles formation, using a constant volume (0.5 mL) of (1 × 10^−3^ M) HAuCl_4_·3H_2_O and different volumes of the prepared stock aqueous solution of *C. olitorius* (mallow) extract at a concentration of (8.0 g/100 mL), with a final concentration of Au NPs of 5 × 10^−4^ M. The appearance of red color proved the formation of the gold nanoparticles due to a complete reduction of Au^+3^ ions, which showed λ_max_ values at 535 nm. The change of the color from yellow to red is a characteristic of the Surface Plasmon Resonance (SPR) of gold nanoparticles different sizes.

The mallow broth extract quantities were varied from 1 to 4 mL. For low amounts of the leaf extract (1–2.2 mL), the Au NPs SPR band displayed low intensity and a broad peak, which suggested a partial reduction of Au^+3^ ions. Upon the addition of higher amounts of the extract (2.4–3.4 mL), the SPR band of the Au NPs shifted to longer wavelengths (red shift), while the red color of the Au NPs solution was established at a λ_max._ value of 535 nm. This red shift indicated that the mean diameter of the NPs decreased, resulting in a spherical shape and a homogeneous distribution of the NPs, as the extract concentration increased. An amount of 3 mL of extract was found to be the optimum quantity for the reduction of Au^+3^ ions (Figure 1). On the other hand, further addition of higher amounts of mallow leaf extract (3.6–4 mL) led to the disappearance of the SPR band of Au NPs, suggesting their agglomeration.

The growth of the Au NPs was also examined using 3 mL of an extract stock solution at a constant concentration (8.0 g/100 mL) with different volumes of (1 × 10^−3^ M) of HAuCl_4_·3H_2_O (Figure S1). The volumes of the gold solution varied between 0.5 and 2 mL. The obtained spectra showed that adding low amounts of HAuCl_4_·3H_2_O (0.5–0.7 mL) caused a gradual increase in the intensity of the SPR band. Further addition of different amounts of HAuCl_4_·3H_2_O (0.8–0.9 mL) resulted in decreased intensity of the SPR band, associated with a red shift to the λ_max_ value of 535 nm. Similar results were reported by Ghosh et al. [32]. This behavior indicated the formation of homogeneous Au NPs with quasi-spherical shapes. On the contrary, the addition of higher amounts of the HAuCl_4_·3H_2_O stock solution (1–2 mL) caused the expansion and almost disappearance of the SPR band around 550 nm, which corresponded to the aggregation of the Au NPs. This behavior could be attributed to the insufficient capping material concentration and, consequently, the incomplete reduction of the Au^+3^ ions. The spectra revealed that 0.5 mL of HAuCl_4_ was the optimum amount for the complete reduction of the Au^+3^ ions.

**Figure 2:**
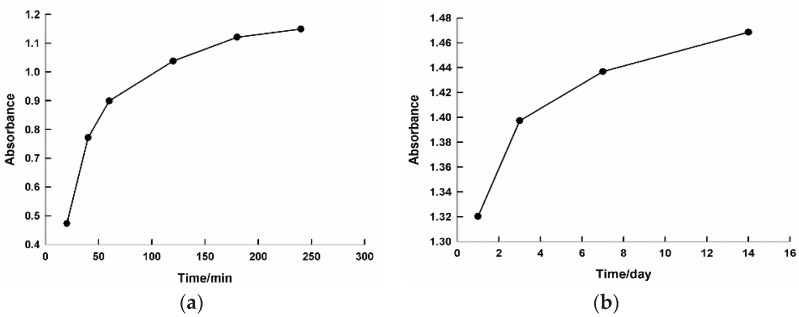
Plot of the intensity of surface plasmon resonance at 535 nm against the reaction time. The time period on the x axis is the difference, graph (**a**) time is by minutes however graph (**b**) is by days.

The reaction between the Au^+3^ ions and the reducing material in the extract was monitored for two weeks in order to examine the stability of the gold NPs. Figure S2 shows the UV–vis spectra of the growing Au NPs as a function of time after the addition of 3 mL of the *C. olitorius* (mallow) leaf extract and 0.5 mL of (0.001 M) HAuCl_4_·3H_2_O. A gradual increase in the intensity of the SPR band was observed by increasing the reaction time, indicating a rise of the concentration of Au NPs. A zigzag pattern was observed after 24 h, which could be attributed to a slight agglomeration of the NPs. Figure 2 illustrates plots of the plasmon intensity at 535 nm against the reaction time. It can be seen that the plasmon intensity gradually increased by increasing the reaction time (Figure 2a). This behavior continued for days up to two weeks (Figure 2b), demonstrating the stability of the synthesized nanoparticles.

**Figure 3:**
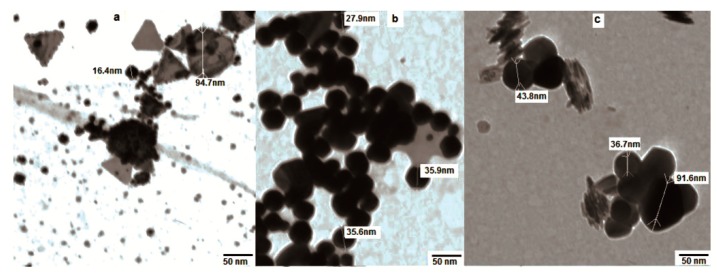
Transmission electron microscopy (TEM) images of Au NPs in the presence of different volumes of the broth extract: (**a**) 2 mL, (**b**) 3 mL, and (**c**) 4 mL.

The transmission electron microscopy (TEM) images clearly revealed the size and shape of the nanoparticles as a function of the extract concentration. A low concentration of the mallow leaf extracts (2 mL) generated Au NPs with predominantly triangular shapes, with some gold nanohexagons. The detected particles were in the range of 16–95 nm (Figure 3a). At this concentration of the extract, the aggregation of the NPs occurred due to the deficiency of capping biomolecules. On the other hand, 3 mL of the mallow extract produced Au NPs with quasi-spherical shapes and an average size of 27–35 nm, in good agreement with the UV–vis spectrum showing the SPR band at 535 nm (Figure 3b). Upon the addition of 4 mL of the mallow extract, the Au NPs exhibited larger pentagonal and hexagonal shapes with an aggregation behavior that led to the disappearance of the SPR band (Figure 3c). Therefore, 3 mL of the mallow extract provided the optimum conditions for the growth of the Au NPs.

**Figure 4:**
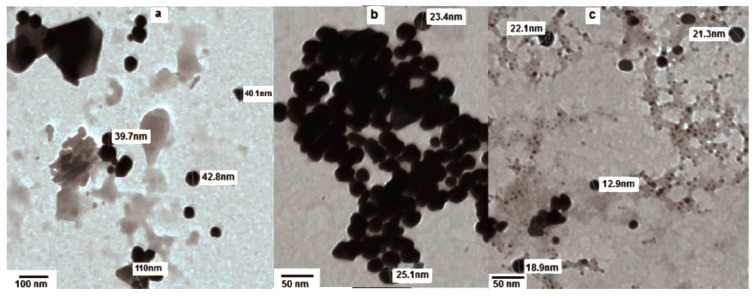
TEM image of Au NPs treated for 1 h with (**a**) 3 mL of extract and 0.7ml HAuCl_4_·3H_2_O and (**b**,**c**) 3 mL of extract and 0.5 mL HAuCl_4_·3H_2_O.

The effect of gold concentrations on the growth of the nanoparticles was also explored. For instance, large heterogeneous shapes and sizes of Au NPs were observed using 0.7 mL of HAuCl_4_·3H_2_O (Figure 4a), while 0.5 mL of the HAuCl_4_·3H_2_O solution yielded homogeneous, quasi-spherical shapes with smaller sizes, ranging from 13 to 25 nm (Figure 4b,c).

The effect of the pH on the formation of Au NPs at SPR = 535 nm was also utilized (Figure S3). It is well known that changing the pH values can alter the electrical charges of biomolecules, which might affect their capping and stabilizing abilities and, subsequently, the growth of the nanoparticles. Our obtained results elucidated that the intensity of the absorption band increased with an increasing pH; however, the λ_max_ values displayed different trends.

For example, in an acidic medium with pH values = 2–3.1, a red shift of λ_max_ was observed. This could be attributed to the partial dissolution of the bioorganic materials of the mallow extract in the acidic medium, which decreased their concentration and the thickness of the layer they formed around the Au NPs, leading to the formation of heterogeneous shapes and sizes of the particles (Figure S4a). A slight blue shift occurred in the pH range 4.6–6.7, and the sharpness of the SPR band increased, which indicated the formation of quasi-spherical Au NPs with smaller particle sizes (Figure S4b). In contrast, a pH between 7 and 8.2 revealed no shift in the Au NPs SPR peak, and the intensity of the absorption band increased, which could be attributed to the formation of NPs with smaller sizes and a homogeneous, quasi-spherical shape. Nevertheless, these Au NPs started to aggregate afterward, causing a decrease in the absorbance band with a blue shift (Figure S4c).

**Figure 5:**
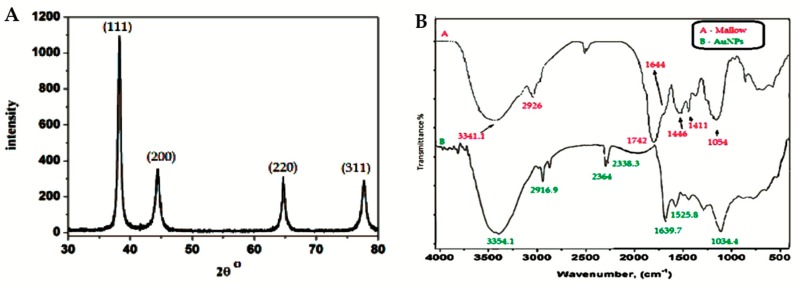
X-ray diffraction (XRD) pattern of Au NPs synthesized using *C. olitorius* (left) and Fourier-transform infrared (FTIR) spectra of (**A**) a plain mallow leaf and (**B**) capped Au NPs (right).

Fourier-transform infrared (FTIR) measurements were performed to identify the potential biomolecules in the *C. olitorius* leaves, which may be responsible for the reduction, capping, and efficient stabilization of the bio-reduced Au NPs. Figure 5 shows the FTIR spectra of the *C. olitorius* leaf extract and its binding to the Au NPs. The FTIR spectra revealed the presence of different functional groups. For instance, Figure 5a displayed peaks at 3341.1 cm^−1^ and 1742 cm^−1^ for the dried *C. olitorius* leaves, which were assigned to the free OH and C=O stretching modes possibly from the 5-caffeoylquinic acid (chlorogenic acid), 3,5-dicaffeoylquinic acid ascorbic acid, α-tocopherol, and quercetin 3-galactoside derivatives (Figure 5 SI) [27,28,29]. Upon binding of the biomolecules to Au NPs, these peaks displayed shifts to 3354 and 1639.7 cm^−1^ for the OH and C=O, respectively (Figure 5b).

The very strong band at 1054 cm^−1^ could be assigned to the C–OH vibrations of the proteins in the *C. olitorius* leaves. This band displayed a shift to 1034 cm^−1^ in Figure 5b due to its binding to the Au NPs. Moreover, the free amine stretching band was observed at 1644 cm^−1^, while, after capping to the Au NPs, this band shifted to 1525.8 cm^−1^, indicating that the proteins in the extract were binding to the Au NPs through the free amine groups as well.

The crystalline structure of the synthesized Au NPs was examined using X-ray diffraction (XRD) measurements. The typical XRD pattern of the Au NPs was observed, as shown in Figure 5. The pattern shows a number of Bragg reflections, which can be classified according to the face-centered cubic (FCC) structure of gold. The diffraction peaks observed at 2θ = 38.31° (111), 44.46° (200), 64.67° (220), and 77.45° (311) are identical to those reported for the standard gold metal (Au^0^) (Joint Committee on powder Diffraction Standards-JCPDS, USA). Hence, the XRD pattern indicates that the Au NPs were essentially crystalline [33,34,35].

It was noticed that the intensity of the peak at the (111) plane had the highest value. The difference of the intensity between the (200) and (111) diffraction peaks (0.32), which was less than the conventional bulk intensity ratio (0.52), indicated that the (111) plane was the predominant orientation [33,34,35]. The average crystal size was calculated according to the Scherrer equation and found to be in the range of 29–37 nm, which is in good agreement with the TEM images.

Thermal gravimetric analysis (TGA) of the prepared Au NPs revealed a steady weight loss in the temperature range of 40–840 °C (Figure S5). The first weight loss observed was at 143 °C and corresponded to the water loss of the capping extract. The thermogram displayed continuous weight loss up to 745 °C and 47.44%, which was most likely a consequence of the surface desorption of the bioorganic compounds present in the nanoparticles powder. Two distinctive weight loss steps were observed in the thermogram in the ranges of 143–401 and 401–745, due to the formation of multiple layers of capping extract around the nanoparticles as well as to the different nature of the extract organic components. The residue was 52.56%, which was attributed to the presence of pure Au NPs after the thermal desorption process. This result was considered an ideal ratio (0.9:1) between the bioorganic materials layer and Au NPs [36].

In-silico tools were exploited to predict the bioactivity, drug-likeness, and ADMET properties of the main components of the *C. olitorius* extract. The bioactivity score profiles of the main components against six different protein structures, namely, G protein-coupled receptors ligand (GPCR), ion channel modulator, kinase inhibitor, nuclear receptor ligand, protease inhibitor, and enzyme inhibitor are shown in Table 1. Most of the selected candidates displayed a bioactivity score of more than 0.00 (positive values indicate greater affinity of the selected drug candidate for the mentioned receptor and enzyme, while negative values mean low affinity); therefore, they can be considered to possess considerable biological activity, especially chlorogenic acid, as all its values tend to be positive.

**Figure 6:**
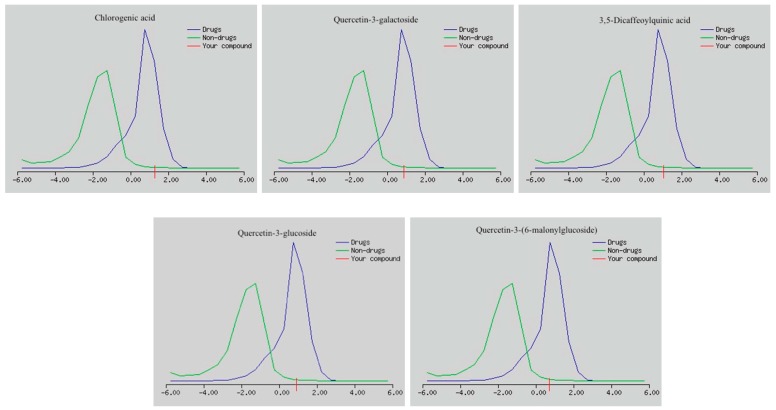
Drug-likeness scores of the main components present in the *C. olitorius* extract.

The drug-likeness scores of the main components is shown in Figure 6 and Table 2. All the screened candidates showed good scores of drug-likeness, ranging from 0.70 to 1.29.

The predicted in vivo ADMET data, i.e., rates of blood–brain barrier (BBB) penetration, percent drug bound to plasma proteins (PPB), human intestinal absorption (HIA%), and Caco-2 and MDCK cell permeability, are shown in Table 2.

The toxicological properties of mutagenicity (Ames zest) and carcinogenicity (mouse and rat) for the selected drug candidates are shown in Table 3. Quercetin-3-galactoside and Quercetin-3-glucoside were predicted to be non-mutagenic by the Ames test. In the prediction of carcinogenicity in mouse and rat, a positive prediction indicated that there was no evidence of carcinogenic activity, i.e., the compound was not carcinogenic in nature, while a negative prediction demonstrated that the compound might exhibit carcinogenic activity.

**Figure 7:**
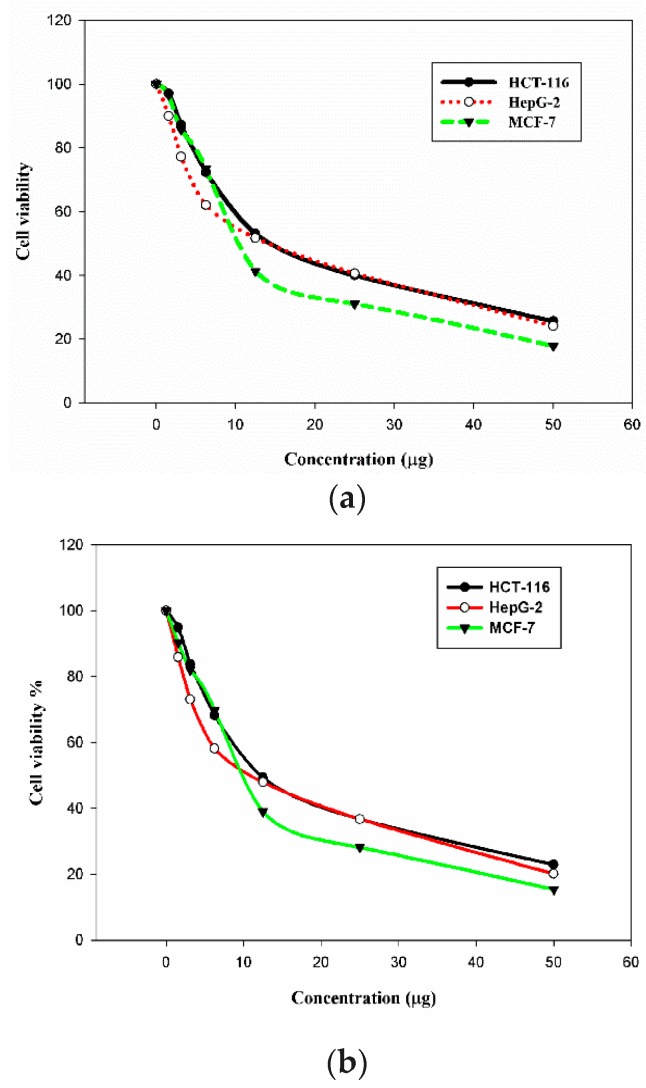
Cytotoxicity of the mallow leaf extract (**a**) and of its bio-functionalized Au NPs (**b**) in HCT-116, HepG-2, and MCF-7 cells.

The in vitro cytotoxic activity of the *C. olitorius* extract and of the bio-functionalized Au NPs was analyzed in three human cancer cell lines: breast adenocarcinoma MCF-7, colon carcinoma HCT-11, and hepatocellular carcinoma HepG-2 cells. The cytotoxic activity was evaluated using the 3-(4,5-dimethylthiazol-2-yl)-2,5-diphenyl tetrazolium bromide (MTT) colorimetric assay [36,37]. Vinblastine was used as a reference cytotoxic compounds in this study. Different concentrations of the Au NPs were applied to the cancer cells, which resulted in a decrease in the viability of the cells. The inhibitory activities of the extract and its bio-functionalized Au NPs are presented in Table 4, Table 5 and Figure 7. Although the mallow extract displayed cytotoxicity in the tested cell lines, its performance was inferior to that of the corresponding functionalized Au NPs. The new bio-functionalized Au NPs exhibited strong to moderate activities in the tested cell lines compared to vinblastine. The best performance of the new Au NPs was in the hepatocellular carcinoma (HepG-2) cells, with an IC_50_ value of 10.3 µg/mL (the IC_50_ of vinblastine in these cells was 9.8 µg/mL). However, the breast adenocarcinoma (MCF-7) and colon carcinoma (HCT-116) cells revealed moderate responses to the Au NPs compared to vinblastine, with IC_50_ = 11.2 and 12.2 µg/mL for MCF-7 and HCT-116, respectively.

## 3. Materials and Methods

Gold (III) chloride.Hydrate HAuCl_4_·3H_2_O was obtained from Sigma-Aldrich chemicals and used as received. A stock solution of HAuCl_4_·3H_2_O (0.01 M) was prepared. Deionizing water was used throughout the reactions. All glass wares were washed with diluted nitric acid HNO_3_ and distilled water. *Corchorus olitorius* (mallow) was purchased from a local market, washed with distilled water to remove any impurities, and dried in a hot oven.

### 3.1. Characterization

The UV absorbance of the synthesized NPs was measured with the single-beam scanning a λ-Helios SP Pye-Unicam spectrophotometer. The phase purity and nature, crystallographic structure, and estimation of the particle size were examined using a “Shimadzu XRD-6000” computerized diffractometer that consists of α-pw1400/90 stabilized X-ray generator, α-pw1050/70 vertical goniometer, α-pw1995/60 proortional counter, and α-pw1930 electronic panel. Nickel-filtered copper radiation with λ = 1.541 Å was utilized in this study. The characterization also included the FTIR analysis of the synthesized Au NPs, by scanning them in the range 400–4000 cm^−1^ at a resolution of 4 cm^−1^. These measurements were carried out on a Nicolet 6700 spectrometer. The growth and morphology of the Au NPs were examined with the JEOL JEM 1200 transmission electron microscopy (TEM), operating at an accelerating voltage of 90 KV. The thermo gravimetric analysis were carried out with a heating rate of 10 °C/min, using a Shimadzu DT-50 thermal analyzer.

### 3.2. Preparation of the Aqueous Extract

AN amount of 8.0 g of dry *C. olitorius* (mallow) leaf was boiled for 15 min, then filtered using Whatmann No.1 filter paper, and brought to 100 mL to obtain the extract. The filtrate used as a reducing agent was kept in the dark at 10 °C and used within one week.

### 3.3. Synthesis of Gold Nanoparticles Using Mallow Leaf Extract

A certain volume of the *C. olitorius* (mallow) leaf extract (1–4) mL was added to a certain volume of HAuCl_4_·3H_2_O solution, and the final volume was adjusted to 10 mL with de-ionized water. The final concentration of Au^0^ was 5 × 10^−4^ M. The solution was stirred for 1 min. The reduction process of Au^+3^ to Au^0^ NPs was followed by the change in color of the solution from yellow to violet to dark pink and red, which depended on several parameters, such as the extract concentration, the gold concentration, and time. The purification of the obtained Au NPs was performed via washing it with ethyl alcohol several times followed by a washing process with deionized water to remove the excess of the extract. The nanomaterials were then dried in an oven at 60 °C for 3–5 h. The nanoparticles were also prepared at different pH values, and the pH of the solutions were adjusted using 0.1 N HCl or 0.1 N NaOH.

### 3.4. In-Silico Predictions

#### 3.4.1. Bioactivity Prediction Using Molinspiration

The main components of the *C. olitorius* extract were evaluated for in-silico bioactivity prediction using the software from Molinspiration Cheminformatics server (http://www.molinspiration.com). Molinspiration offers a wide range of cheminformatics software tools, like SMILES and SD file conversion, allowing the calculation of diverse molecular properties required for QSAR studies, molecule processing and manipulation, molecular modelling and drug design, along with supporting virtual screening which is fragment-based, the prediction of bioactivity, and the visualization of the data obtained. The Molinspiration tools are written in Java, so they can be practically used on any computer platform. Molinspiration calculates the bioactivity contribution of each substructural fragment on the basis of the fragment’s basic property. The chemical structures of the main components of the *C. olitorius* extract were drawn directly into the designated window. The calculated sum of activity contributions of the fragments was used in predicting the bioactivity score (a numerical value, typically between −3 and 3) of these molecules. Molecules with the highest activity score have the highest probability to be active.

The above described protocols were employed, and screening models were developed for four important classes of drugs, i.e., kinase inhibitors, G protein-coupled receptors ligand (GPCR ligands), nuclear receptor ligands, and ion channel blockers or modulators. In addition, enzyme and protease inhibitions were also performed.

#### 3.4.2. Drug-Likeness Prediction Using Molsoft

The same components were evaluated for drug-likeness score predictions using the software from the Molsoft server (http://www.molsoft.com). MolSoft extends its services and software tools for structure predictions. This helps in interpreting the spatial organization of drug candidates, how they interact with each other, their biological substrates and drug-like compounds at the atomic level by implementing some rules and algorithms for specific biomedical problems. Drug-likeness is a qualitative approach used for determining the drug-like properties of a compound. It consists of a complex balance of assorted molecular and structural properties that play a crucial role in determining whether the specific drug candidate is similar to known drugs or not.

#### 3.4.3. ADMET Prediction Using PreADMET

The same components were subjected to ADMET predictions using the software from the PreADMET server (http://www.preadmet.com). The molecular structure of the investigated molecules plays a key role in determining the ADMET properties and their pharmacokinetic behavior. In order to find if the selected compounds would be able to pass across the blood–brain barrier, Blood–Brain Barrier (BBB) penetration prediction was carried out, a crucial investigation in the pharmaceutical sphere. Central Nervous System (CNS)-active molecules are capable of passing across the BBB, while CNS-inactive compounds do not and do not have CNS side effects [38]. Generally, only an unbound drug can be transported or can diffuse through cell membranes and interac with pharmacological targets. A drug’s action, together with its efficacy and disposition, is influenced by its binding to plasma proteins, which in turn makes the PPB% an important pharmacokinetic factor [39]. Predicting human intestinal absorption (HIA%) is also considered crucial in the development of potential drug candidates. The ratio of excretion or cumulative excretion in bile, urine, and feces is used to evaluate the sum of bioavailability and absorption as HIA% [40]. Oral bioavailability is considered a noteworthy feature for the identification of bioactive compounds. For the prediction of the oral absorption of drugs, models like the Madin–Darby canine kidney (MDCK) and Caco-2 cells are considered well-grounded in vitro models. Caco-2 cells are well-differentiated intestinal cells derived from a human colorectal carcinoma. They possess many of the morphological and functional properties of intestinal epithelial cells in vivo [41]. MDCK cells are preferable to Caco-2 cells as their growth rate is higher than that of Caco-2 cell. The MDCK cells model, thus, may be utilized as a route for rapidly screening permeability [42]. The scrutiny of toxicity is a vital feature in the conception of drugs, as it can foretell the mutagenicity and carcinogenicity of new compounds. The Ames test is a simple method to predict the mutagenicity of a compound [43]. Carcinogenicity is a type of toxicity which can lead to the growth of cancer in the body. The carcinogenicity test typically uses rats or mice exposed to a molecule or drug candidate. The properties of mutagenicity and carcinogenicity are established by the National Toxicology Program (NTP) and the USA Food and Drug Administration (FDA), on the basis of results from in vivo tests in mice and rats obtained over at least 2 years.

### 3.5. Biological Tests

#### 3.5.1. Cell Culture

The tumor cell lines breast adenocarcinoma (MCF-7), human colon carcinoma (HCT-116), and hepatocellular carcinoma (HepG-2) were obtained from the American Type Culture Collection (ATCC, Rockville, MD, USA). The cells, which were grown in the presence of 10% inactivated fetal calf serum and 50 µg/mL gentamycin, were cultivated in RPMI-1640 medium at a temperature of 37 °C in a humidified atmosphere with 5% CO_2_ and were sub-cultured two to three times a week.

#### 3.5.2. Cytotoxic Activity Assessment Using a Viability Assay

The tumor cell lines were suspended in medium at a concentration of 5 × 10^4^ cell/well in Corning^®^ six-well tissue culture plates and were incubated for 24 h. The tested Au NPs, with concentrations ranging from 0 to 50 μg/mL, were then added into the six-well plates (six replicates) to achieve different NPs concentrations. Six vehicle controls with media or 0.5% DMSO were set up for each six-well plate as a control. After being incubated for 24 h, the numbers of viable cells were determined by the MTT test. Briefly, the media was removed from the six-well plates and replaced with 100 μL of fresh culture RPMI 1640 medium without phenol red, then 10 µL of the 12 mM MTT stock solution (5 mg of MTT in 1 mL of PBS) was added to each well, including the untreated controls. The six-well plates were then incubated at 37 °C and 5% CO_2_ for 4 h. An 85 μL aliquot of the media was removed from the wells, and 50 µL of DMSO was added to each well and mixed thoroughly with a pipette, and the sample was incubated at 37 °C for 10 min. Afterwards, the optical density was measured at 590 nm with a microplate reader (SunRise, TECAN, Inc., Baldwin Park, CA, USA) to determine the number of viable cells; the percentage of cell viability was calculated as [1 − (ODt/ODc)] × 100%, where ODt is the mean optical density of the wells treated with the tested compound, and ODc is the mean optical density of the untreated cells. The relation between surviving cells and drug concentration was plotted to obtain the survival curves of each tumor cell line treated with the NPs. The 50% inhibitory concentration (IC_50_), i.e., the concentration required to cause toxic effects in 50% of intact cells, was estimated from the dose response curves for each concentration, using the Graph pad Prism software (San Diego, CA, USA) [36]. Vinblastine was used as a reference drug in this study.

## 4. Conclusions

In conclusion, we have presented a one-step green synthesis of gold nanostructures using naturally occurring biodegradable plant-based surfactants from *C. olitorius* leaves, without any special reducing or capping agents. Upon the addition of the gold solution to the plant extract, the color changed from colorless to red, with the SPR band at 535 nm, signifying the formation of Au NPs. The size and shapes of the obtained nanoparticles were manipulated by varying the ratio of the broth extract and the metal solution and were visualized using TEM. X-ray diffraction was used to confirm the crystalline nature of the nanoparticles. The obtained biosynthesized nanoparticles exhibited stability up to two weeks. The predictive analyses revealsedthat the main constituents of this plant extract have moderate to good bioactivity and drug-likeness scores. The ADMET studies were also undertaken to predict the pharmacokinetic behavior of these main components, thus supporting a future drug development program for drugs with increased effectiveness and decreased toxicity. Moreover, these synthesized particles displayed strong to moderate cytotoxic activities against three cancer cell lines (HCT-116, HepG-2, and MCF-7). It is important to note that, in spite of other reports, this methodology shows the successful isolation of nanoparticles at ambient temperature, which allowed us to utilize the richness of the plant extract without any degradation of its components. In addition, it enhanced the cytotoxicity of the obtained nanoparticles in the tested cancer cell lines, as a result of the combination of the nanoparticles with the plant extract.

## Figures and Tables

**Table 1:** Bioactivity of the main components present in the *C. olitorius* extract.

Compound Name	GPCR Ligand	Ion Channel Modulator	Kinase Inhibitor	Nuclear Receptor Ligand	Protease Inhibitor	Enzyme Inhibitor
Chlorogenic acid	0.29	0.14	0	0.74	0.27	0.62
Quercetin-3-galactoside	0.06	−0.04	0.13	0.20	−0.06	0.42
3,5-Dicaffeoylquinic acid	0.18	0.03	−0.02	0.46	0.13	0.37
Quercetin-3-glucoside	0.07	−0.11	0.08	0.01	−0.07	0.47
Quercetin-3-(6-malonylglucoside)	−0.62	−1.50	−1.03	−0.98	−0.40	−0.66

**Table 2:** ADME (absorption, distribution, metabolism, excretion) properties and drug-likeness scores of the main components present in the *C. olitorius* extract.

Compound Name	BBB ^a^	PPB ^b^	HIA ^c^	Caco-2 ^d^	MDCK ^e^	Drug-Likeness Scores
Chlorogenic acid	0.034	41.96	20.43	18.72	4.51	1.29
Quercetin-3-galactoside	0.032	59.16	11.78	9.44	2.49	0.89
3,5-Dicaffeoylquinic acid	0.035	86.06	23.12	19.32	0.04	1.05
Quercetin-3-glucoside	0.032	58.16	11.78	4.49	2.21	0.91
Quercetin-3-(6-malonylglucoside)	0.047	35.48	0.39	6.70	0.06	0.70

^a^ blood–brain barrier penetration, ^b^ plasma protein binding, ^c^ human intestinal absorption, ^d^ Caco-2 cell permeability, ^e^ MDCK cell permeability.

**Table 3:** Toxicity prediction of the main components present in the *C. olitorius* extract.

Compound Name	Ames Test Mutagenicity	Mouse Carcinogenicity	Rat Carcinogenicity
Chlorogenic acid	Mutagenic	Positive	Negative
Quercetin-3-galactoside	Non-Mutagenic	Negative	Negative
3,5-Dicaffeoylquinic acid	Mutagenic	Positive	Positive
Quercetin-3-glucoside	Non-Mutagenic	Negative	Negative
Quercetin-3-(6-malonylglucoside)	Mutagenic	Positive	Negative

**Table 4:** Cytotoxicity of the mallow leaf extract in HCT-116, HepG-2, and MCF-7 cells, with IC_50_ = 12.2, 10.3, and 11.2 µg/mL, respectively.

Sample Conc. (μg/mL)	HCT-116 Viability %		HepG-2 Viability %		MCF-7 Viability %	
	Stand.	Sample	Stand.	Sample	Stand.	Sample
50	12.16	25.61	15.38	24.07	7.82	17.80
25	15.54	39.92	27.35	40.59	15.18	31.02
12.5	18.92	53.06	43.59	51.67	29.26	41.22
6.25	39.86	72.34	53.85	61.99	42.35	73.43
3.125	47.30	87.11	69.23	77.20	56.54	85.64
1.56	58.11	97.01	76.82	89.91	67.24	96.51
0	100	100	100	100	100	100

**Table 5:** Cytotoxicity of Au NPs in HCT-116, HepG-2, and MCF-7 cells, with IC_50_ = 12.2, 10.3, and 11.2 µg/mL, respectively.

Sample Conc. (μg/mL)	HCT-116 Viability %		HepG-2 Viability %		MCF-7 Viability %	
	Stand.	Sample	Stand.	Sample	Stand.	Sample
50	12.16	22.96	15.38	20.13	7.82	15.4
25	15.54	36.73	27.35	36.74	15.18	28.17
12.5	18.92	49.34	43.59	47.91	29.26	38.98
6.25	39.86	68.22	53.85	58.14	42.35	69.73
3.125	47.30	83.74	69.23	73.06	56.54	81.97
1.56	58.11	94.85	76.82	85.82	67.24	90.29
0	100	100	100	100	100	100

## Supplementary Materials

Supplementary materials can be found at http://www.mdpi.com/1422-0067/19/9/2612/s1.
