# Supplementary material for: Successful Green Synthesis of Gold Nanoparticles using a Corchorus olitorius Extract and Their Antiproliferative Effect in Cancer Cells
Source: Int J Mol Sci. 2018 Sep 3;19(9):2612. doi: 10.3390/ijms19092612 (PMC6163711; doi:10.3390/ijms19092612)
Supplement: Supplementary file 1 [file ijms-19-02612-s001.pdf]

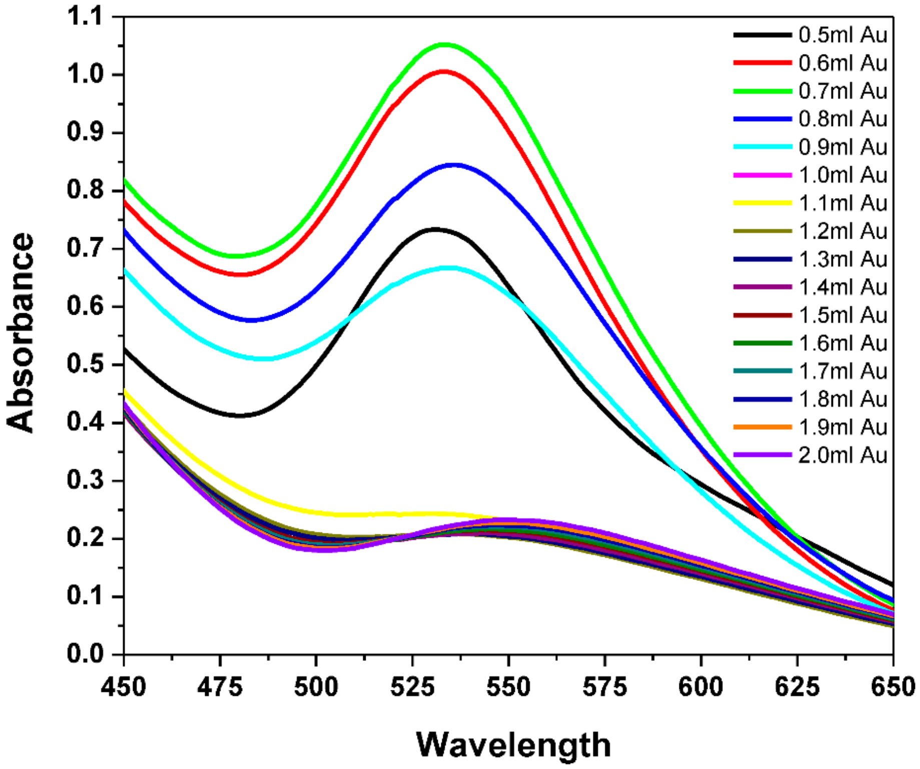

Figure S1. UV-visible spectra of gold nanoparticles at different concentration of metal ions.

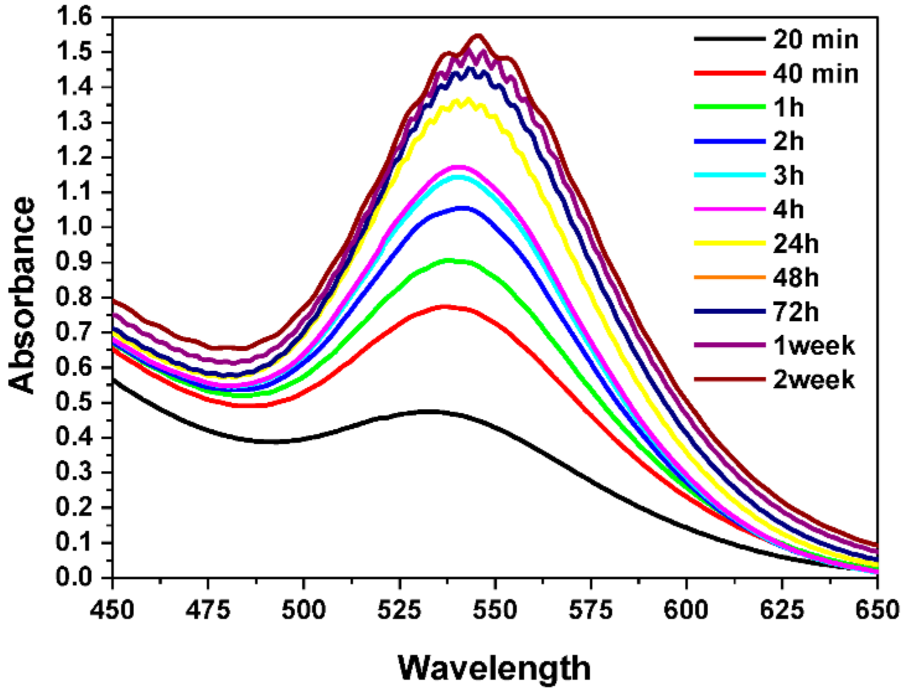

Figure S2. UV-visible spectra of Au NPs as a function of time at room temperature.

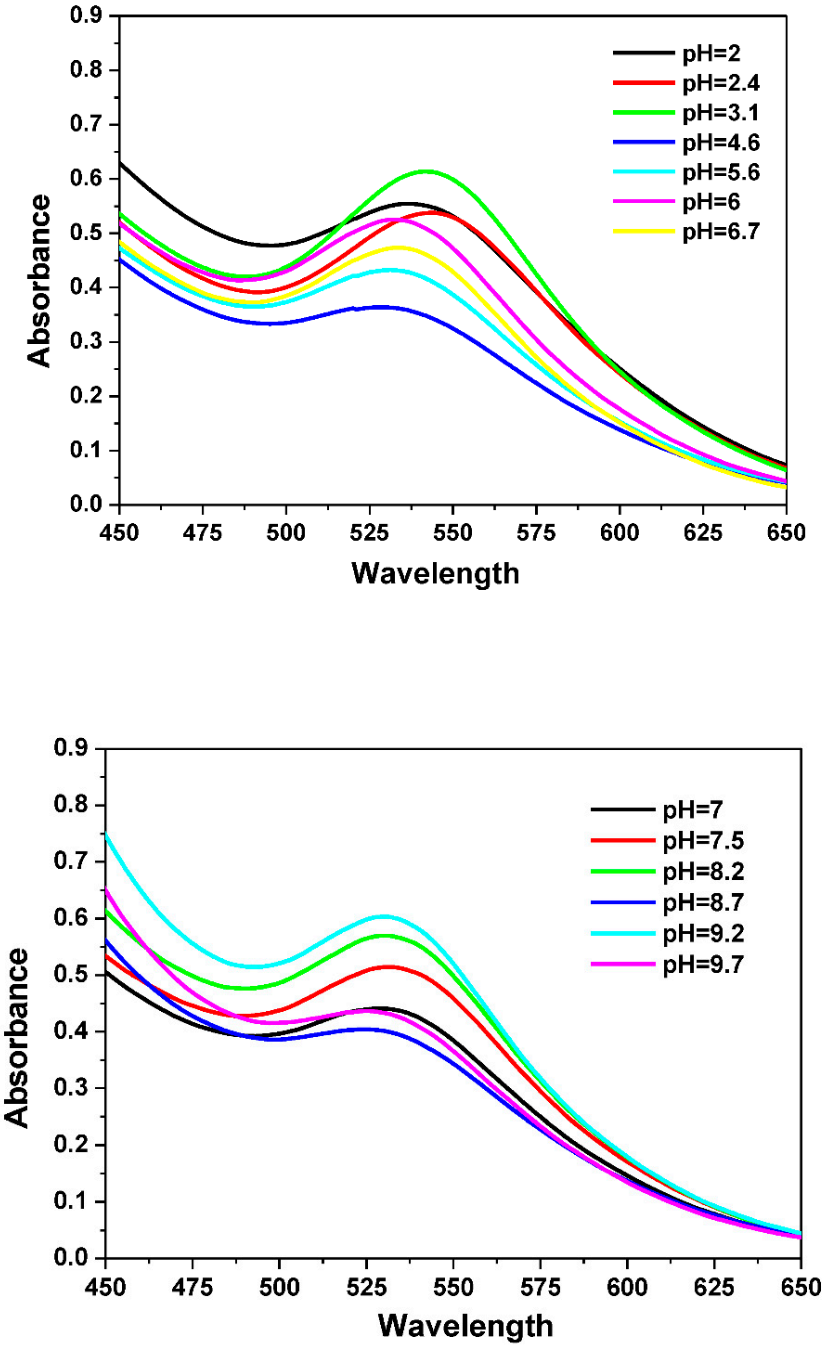

Figure S3. Effect of pH on the formation of Au NPs.

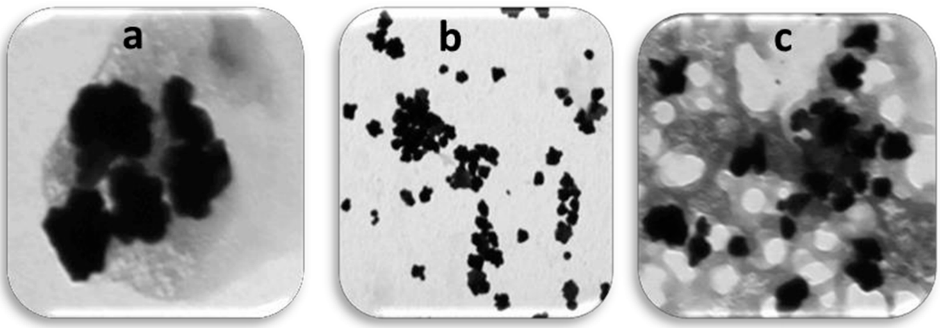

Figure S4. TEM images of Au NPs at (A) pH = 3.1 (B) pH = 6.7 and (c) pH = 9.7.

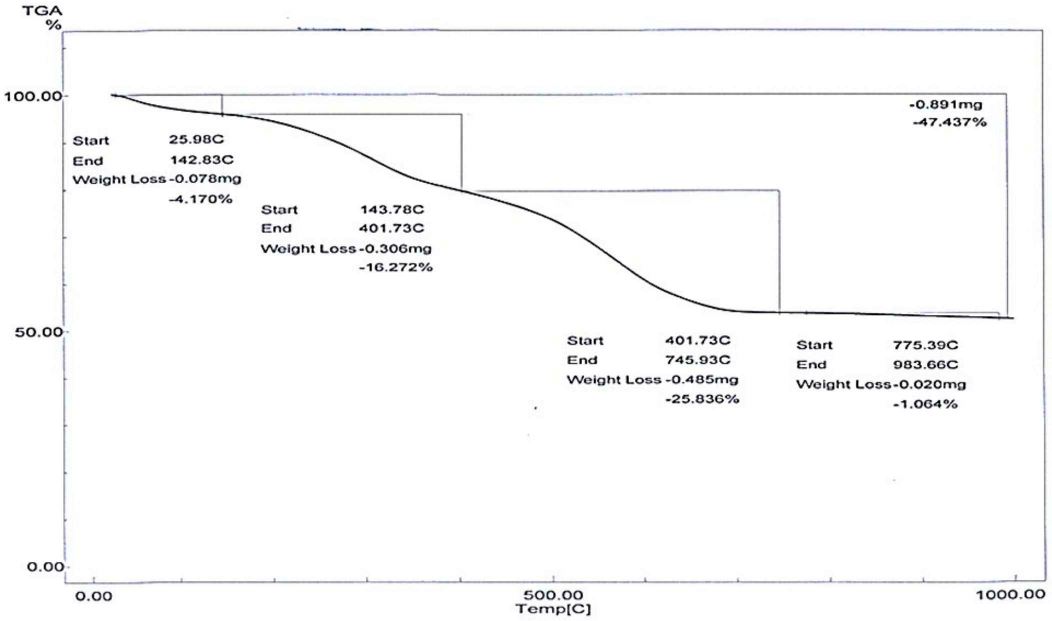

Figure S5. TGA thermogram of capped Au NPs prepared using mallow leave extract.

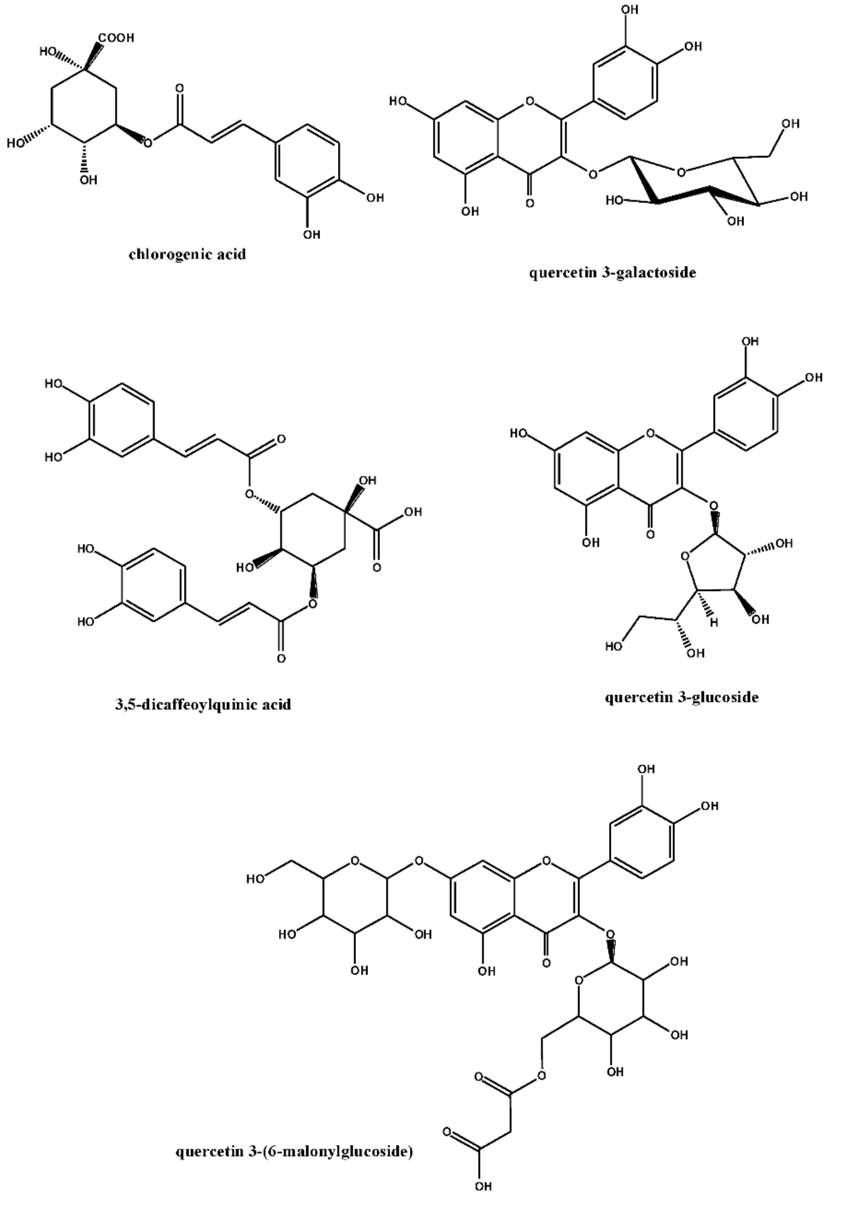

Figure S6. Main components of *Corchorus olitorius.*
